# Biphenyl 2,3-Dioxygenase in *Pseudomonas alcaliphila* JAB1 Is Both Induced by Phenolics and Monoterpenes and Involved in Their Transformation

**DOI:** 10.3389/fmicb.2021.657311

**Published:** 2021-04-30

**Authors:** Andrea Zubrova, Klara Michalikova, Jaroslav Semerad, Michal Strejcek, Tomas Cajthaml, Jachym Suman, Ondrej Uhlik

**Affiliations:** ^1^Department of Biochemistry and Microbiology, Faculty of Food and Biochemical Technology, University of Chemistry and Technology, Prague, Czechia; ^2^Institute of Microbiology, Academy of Sciences of the Czech Republic, Prague, Czechia; ^3^Faculty of Science, Institute for Environmental Studies, Charles University, Prague, Czechia

**Keywords:** secondary plant metabolites, aromatic ring-hydroxylating dioxygenases, biphenyl dioxygenase, phenolics, monoterpenes

## Abstract

The involvement of bacterial aromatic ring-hydroxylating dioxygenases (ARHDs) in the degradation of aromatic pollutants, such as polychlorinated biphenyls (PCBs), has been well studied. However, there is considerable speculation as to the origin of this ability. One hypothesis is centered on a connection between the ability to degrade aromatic pollutants and the necessity of soil bacteria to cope with and/or utilize secondary plant metabolites (SPMs). To investigate this connection, we researched the involvement of biphenyl 2,3-dioxygenase (BPDO), an ARHD essential for the degradation of PCBs, in the metabolism of SPMs in the soil bacterium *Pseudomonas alcaliphila* JAB1, a versatile degrader of PCBs. We demonstrated the ability of the strain JAB1 to transform a variety of SPMs, namely the flavonoids apigenin, flavone, flavanone, naringenin, fisetin, quercetin, morin, and catechin, caffeic acid, *trans*-cinnamic acid, and the monoterpenes (*S*)-limonene and (*R*)-carvone. Of those, the transformation of flavone, flavanone, and (*S*)-limonene was conditioned by the activity of JAB1-borne BPDO and thus was researched in more detail, and we found evidence for the limonene monooxygenase activity of the BPDO. Furthermore, the *bphA* gene in the strain JAB1 was demonstrated to be induced by a wide range of SPMs, with monoterpenes being the strongest inducers of the SPMs tested. Thus, our findings contribute to the growing body of evidence that ARHDs not only play a role in the catabolism of aromatic pollutants, but also of natural plant-derived aromatics, and this study supports the hypothesis that ARHDs participate in ecological processes mediated by SPMs.

## Introduction

Secondary plant metabolites (SPMs) are a highly diverse group of compounds of plant origin with numerous functions in ecological processes. Among other roles, SPMs act as allelopathic chemicals, agents that protect the plant from pathogens and herbivores, and are involved in the plant’s protection against abiotic stress, in nutrient acquisition, attraction of pollinators, etc. (Rausher, 2001; Cesco et al., 2012; Böttger et al., 2018). SPMs also represent a significant proportion of rhizodeposits, i.e., plant-derived organic matter released into soil (Dennis et al., 2010; Leewis et al., 2016). The SPMs released into soil include monoterpenes and a variety of phenylpropanoid pathway-synthesized phenolics, which can be further divided into acetophenones, phenolic acids, flavonoids, lignins and lignans, tannins, xanthones, etc. (Vuolo et al., 2019).

SPMs have a wide range of both positive and negative effects on soil microbial communities (reviewed by Musilova et al., 2016). They can serve as growth substrates for many bacteria (Donnelly et al., 1994; Fraraccio et al., 2017) or are involved in plant-microbe crosstalk during the establishment of mutualistic symbiosis (Liu and Murray, 2016). On the other hand, they can exert antimicrobial activity (Singer et al., 2003) or disrupt bacterial quorum sensing (Koh et al., 2013). As a result, due to the presence of various SPMs, the rhizosphere becomes a highly selective environment (Kim et al., 1995; Dakora and Phillips, 1996; Xu and Lee, 2001). To deal with the selective pressure resulting from the detrimental effect of SPMs, soil microbiota responds with two mechanisms of resistance. The first is the exclusion of toxic compounds *via* transmembrane efflux pumps (Parniske et al., 1991; Palumbo et al., 1998; González-Pasayo and Martínez-Romero, 2000; Papadopoulos et al., 2008; Martinez et al., 2009); the other is the enzymatic transformation of a SPM to a non-toxic form or its mineralization, which provides the further benefit of a carbon and/or energy yield (Shaw et al., 2006; Marmulla and Harder, 2014). One group of enzymes involved in the bacterial transformation of aromatic compounds is aromatic ring-hydroxylating dioxygenases (ARHDs), multicomponent non-heme oxidoreductases that usually exhibit broad substrate specificity (Baldwin et al., 2000). ARHDs have been mostly described in connection with the degradation of aromatic pollutants, including polychlorinated biphenyls (PCBs), under aerobic conditions (Eltis and Bolin, 1996; Vezina et al., 2008); they attack aromatic compounds, which are usually inert and stable, and activate the substrate for subsequent reactions leading to the cleavage of the aromatic ring (Parales and Resnick, 2006).

The chemical structures of a variety of SPMs, such as phenolics, are remarkably similar to those of aromatic pollutants (Singer et al., 2003). This resemblance led to the hypothesis that broad-substrate-specificity biodegradative enzymes, such as ARHDs, that were identified to degrade aromatic pollutants originally evolved in bacteria to provide them with the ability to degrade SPMs (Donnelly et al., 1994; Focht, 1995; Hernandez et al., 1997; Singer et al., 2003; Furukawa et al., 2004). An example supporting this hypothesis is the enzymes of the biphenyl/PCB degradation pathway (*bph*); although the presence of biphenyl is not common in soils, biphenyl-degrading bacteria are ubiquitous, even in pristine soils and sediments (Focht, 1995). Additionally, bacteria efficiently degrading PCBs have been isolated from termite guts, where a lignin-rich diet is decomposed (Chung et al., 1994). More recently, Sylvestre and colleagues concluded that the biphenyl degradation pathway in soil bacteria may have evolved to serve different ecological functions, including the metabolism of SPMs (Toussaint et al., 2012; Pham et al., 2015). These studies, however, only provide a partial understanding of how SPMs are linked with biodegradative enzymes with broad substrate specificities, including ARHDs.

**Figure 1:**
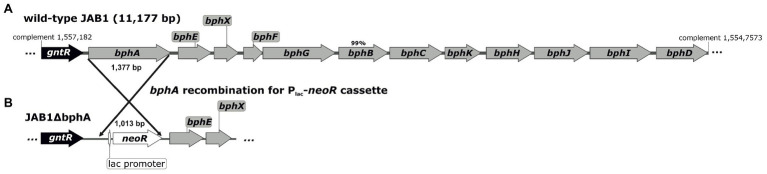
Architecture of the biphenyl/PCB degradation pathway (*bph* operon) in the wild-type JAB1 **(A)** and in the *bphA*-null mutant JAB1ΔbphA **(B)**. The position of the *bph* operon in the JAB1 annotated whole genome sequence (Genbank accession no. CP016162.1) is indicated. The deduced protein sequences encoded by all genes shown were 100% identical with their homologues from *Pseudomonas furukawaii* KF707, except BphB with the identity score of 99%. The gene *gntR* encodes for a putative GntR-family transcription regulator; *bphA* and *bphE* – large and small subunit of the two-component biphenyl dioxygenase, respectively; *bphX* – protein with unknown function; *bphF* and *bphG* – ferredoxin and ferredoxin reductase, respectively; *bphB* – *cis*-2,3-dihydrobiphenyl-2,3-diol dehydrogenase; *bphC* – 2,3-dihydroxybiphenyl-1,2-dioxygenase; *bphK* – glutathione S-transferase; *bphH* – 2-hydroxypenta-2,4-dienoate hydratase; *bphJ* – acetaldehyde dehydrogenase (acetylating); *bphI* – 4-hydroxy-2-oxovalerate aldolase; and *bphD* – 2-hydroxy-6-oxo-6-phenylhexa-2,4-dienoate (HOPDA) hydrolase.

In this study, we worked under the hypothesis that ARHDs might have originally evolved for the degradation/detoxification of SPMs, and that they were, due to their broad substrate specificity, exapted for the degradation of aromatic pollutants. Thereby, the SPMs may act as (i) substrates for ARHDs and (ii) efficient inducers of the ARHD genes. With the example of biphenyl 2,3-dioxygenase (BPDO) from the soil PCB-degrader *Pseudomonas alcaliphila* JAB1 (Ryslava et al., 2003; Ridl et al., 2018), we provide a complex investigation of how a wide variety of SPMs, including flavonoids, phenolic acids, coumarins, and monoterpenes, induce the expression of this BPDO, and that this BPDO is involved in the degradation of some of these SPMs. BPDO is an exemplary ARHD, which catalyzes the first step of biphenyl degradation pathway and has a key role in substrate specificity of the whole pathway (Mondello et al., 1997). It is a multicomponent enzyme that consists of a terminal dioxygenase and an electron transfer chain. The terminal dioxygenase comprises large subunits (α) with a substrate-binding domain and small subunits (β), encoded by *bphAE* genes (also known as *bphA1A2* in the literature). The electron transfer chain contains ferredoxin genes (encoded by *bphF* or *bphA3*), and ferredoxin reductase genes (encoded by *bphG* or *bphA4*; Furukawa and Fujihara, 2008; Kumar et al., 2011; Pham et al., 2012). In the strain JAB1, these genes are encoded within the chromosome-borne cluster *bphAEXFGBCKHJID*, the organization of which is identical to that from *Pseudomonas furukawaii* KF707 (Figure 1). Moreover, the deduced protein sequences encoded by the JAB1-borne *bph* genes are >99% identical with their KF707 orthologs (Watanabe et al., 2000; Furukawa and Fujihara, 2008; Ridl et al., 2018). Our results contribute to the knowledge on the role of ARHDs in the SPM-mediated plant-microbe interactions occurring in soil and broaden our understanding of soil ecology.

## Materials and Methods

### Bacterial Strains, Culture Media, and Growth Conditions

The bacterial strain *P. alcaliphila* JAB1 was isolated from legacy PCB-contaminated soil (Ryslava et al., 2003) and has been shown to be a versatile degrader of PCBs (Ridl et al., 2018). A JAB1 *bphA*-null mutant with non-functional BPDO, designated JAB1ΔbphA (Figure 1), was constructed in this study as described further. *Escherichia coli* DH11S was used for the purposes of plasmid cloning and manipulation (Lin et al., 1992).

Lysogeny broth (LB) medium (Sigma-Aldrich, United States) and mineral salt solution (MSS; Uhlik et al., 2012) were used for the cultivation of the JAB1 strain, the latter amended with a carbon source: pyruvate (Sigma-Aldrich, United States), biphenyl (Sigma-Aldrich, United States), or individual SPMs. Solid MSS-based medium was solidified with 2% agar (w/v), and biphenyl as a sole carbon source was provided as vapors from biphenyl crystals deposited in the lid of the Petri dish. Media for the cultivation of the strains JAB1ΔbphA and JAB1/pUCP18-RedS-catR were amended with the antibiotics kanamycin (10 mg.L^−1^) and chloramphenicol (300 mg.L^−1^), respectively. Liquid cultures were grown at 28°C on a rotary shaker at 130 RPM. Bacterial strains were maintained in glycerol stock (25% v/v) at −80°C and revived on LB plates. *Escherichia coli* transformants were selected and cultivated on LB plates amended with ampicillin (150 mg.L^−1^) or chloramphenicol (25 mg.L^−1^).

### Chemicals

**Figure 2:**
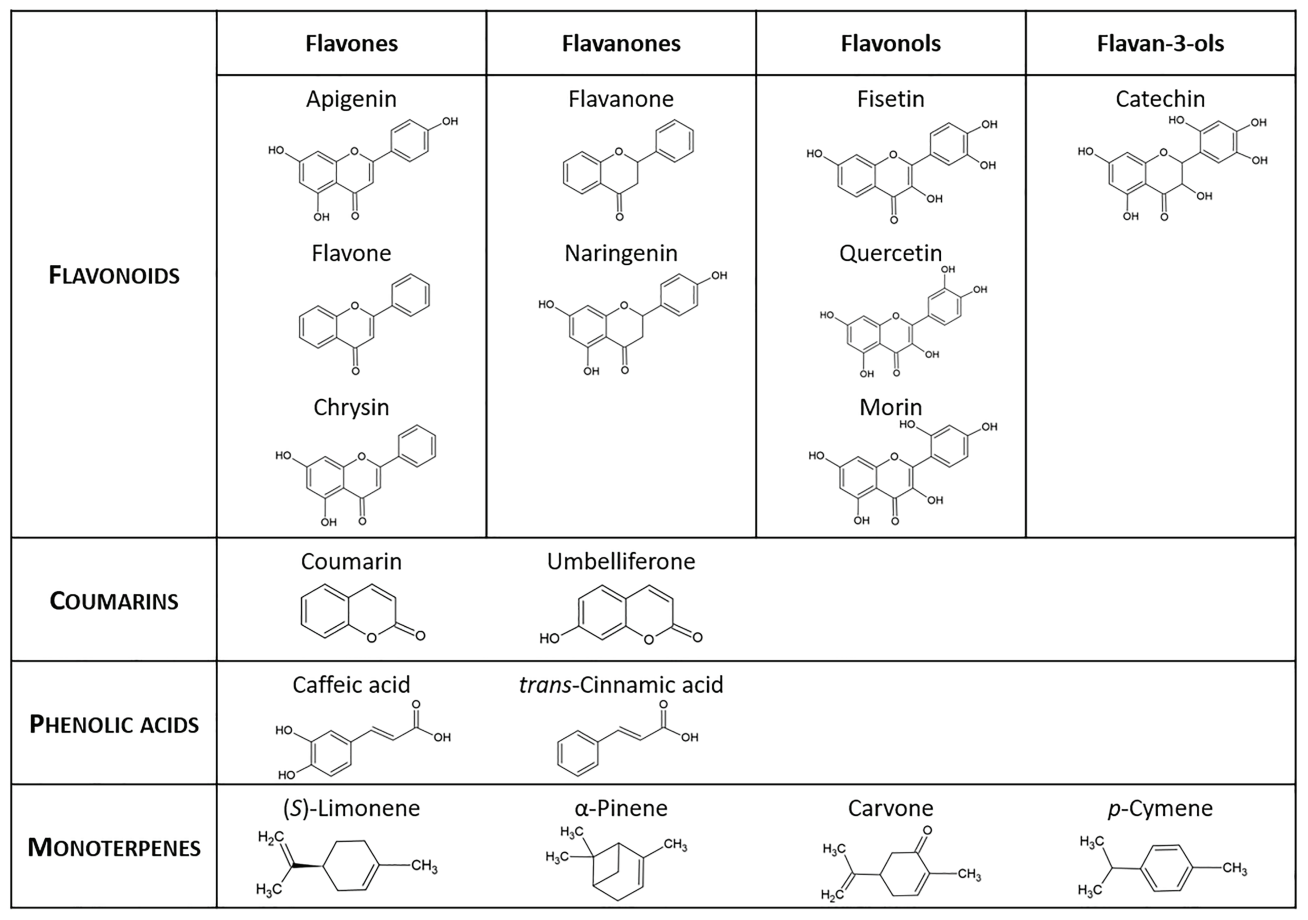
Secondary plant metabolites used in this study.

Biphenyl and SPMs (Figure 2) were purchased from Sigma-Aldrich. Stock solutions of biphenyl and SPMs were prepared in molecular biology-grade ethanol (VWR Chemicals, Unites States), except for quercetin and morin that were prepared immediately prior to use by dissolving in ethanol and alkalization by KOH, and fisetin and chrysin that were dissolved in ethanol:dimethyl sulphoxide (DMSO; 1:1, v/v). Stock solutions were sterilized by filtration with a pore size of 0.2 μm (GD/X PES Filter Media, Whatman™) and stored at 5°C in the dark.

### Utilization of SPMs by the Strain JAB1

The ability of the strain JAB1 to utilize the SPMs listed in Figure 2 as a sole carbon source was monitored by measuring OD_600_ in liquid MSS. See Supplementary Material for details.

### Construction of the JAB1 *bphA*-Null Mutant

To determine the contribution of the JAB1-borne BPDO activity to the transformation of SPMs, the JAB1-derivative JAB1ΔbphA with a deleted *bphA* gene was prepared (Figure 1). For this purpose, the l-arabinose-inducible lambda Red recombinase system was employed, borne on the plasmid pUCP18-RedS, which enables site-specific homologous recombination in *Pseudomonas*. The methodology was adopted from Lesic and Rahme (2008) with modifications as described in Supplementary Material.

### Transformation of SPMs by the Strain JAB1

To test the ability of the JAB1 strain to transform the SPMs listed in Figure 2 and determine the contribution of the BPDO, a resting cell assay (RCA) was employed as described in Vergani et al. (2019) and Garrido-Sanz et al. (2020). The strains JAB1 and JAB1ΔbphA were cultivated O/N in LB, the latter amended with kanamycin (10 mg.L^−1^). Cells were harvested by centrifugation, washed twice in 0.85% NaCl, and adjusted to an OD_600_ = 1 with MSS. For the low-volatile compounds (flavonoids, phenolic acids, and coumarins, Figure 2), 5 ml aliquots of washed cell suspensions were distributed into 50 ml polypropylene tubes amended with a substrate from which ethanol was evaporated, to yield a final concentration of 0.25 mM. For the terpenoids (Figure 2), the RCA was performed in 50 ml glass vials without previous evaporation of the solvent to avoid substrate loss. Each combination of a SPM and a bacterial strain was prepared in triplicate; the abiotic control was MSS with the added substrate without cells. Samples were incubated for 24 h at 28°C/130 rpm and then stored at −80°C until further analysis (see Supplementary Material for details). The depletions rates of SPMs were expressed as *R* = (SPM content in the presence of a respective strain)/(SPM content in the abiotic control sample). The calculation of standard deviations (SD) of resulting *R* values was based on the error propagation according to Tellinghuisen (2001). The depletion of SPMs and the degradation products of flavone, flavanone, and (*S*)-limonene were analyzed by liquid and gas chromatography coupled with mass spectrometry, for details of the analyses see Supplementary Material. The obtained depletion rates of each SPM were analyzed by the single factor ANOVA (*p* < 0.05) and Tukey-Kramer *post-hoc* test.

### Induction of the *bphA* Gene in the Strain JAB1 by SPMs

The ability of SPMs (Figure 2) to induce the *bphA* gene in JAB1 was determined as the level of *bphA* transcripts in JAB1 cells upon incubation with individual SPMs, using quantitative reverse transcription-PCR (RT-qPCR) and cDNA obtained from JAB1’s total RNA as the template and the 16S RNA gene as the reference gene.

Briefly, JAB1 cells from the starting culture in LB medium were harvested, washed, and resuspended to OD_600_ = 0.025 in MSS amended with 30 mM of sodium pyruvate. This suspension (0.5 l) in a 2 l Erlenmayer flask was grown for ca 9 h at 28°C/130 RPM until the mid-log phase was reached (OD_600_ 0.1–0.2). Cells were harvested by centrifugation, washed in 0.85% NaCl, and concentrated to OD_60_ = 1 in MSS. Aliquots of cell suspension (10 ml) were distributed into 100 ml Erlenmayer flasks amended with 0.25 mM of an individual SPM or biphenyl with the ethanol evaporated prior to the addition of the cell suspension. Bacterial culture with no addition was used as a control. Cells were incubated for 1 and 3 h at 28°C/130 RPM. All the samples were prepared in four replicates. After co-incubation, the cells from 2 ml of each culture were pelleted by centrifugation (10 min, 7,000 RCF at 4°C), the resultant cell pellets were stored at −80°C.

The total RNA was isolated using a Qiagen RNAsy Mini Kit according to the manufacturer’s protocol, slightly modified as follows. The enzymatic lysis of pelleted cells was performed in 300 μl of TE buffer amended with 1 mg/ml of lysozyme incubated for 10 min at room temperature on a benchtop shaker. RLT buffer (400 μl) amended with 10 μl/ml ß-mercaptoethanol was added to the reaction mix. After the addition of 500 μl of molecular biology-grade ethanol, the precipitate formed was removed from microtubes with the end of a pipette tip. The whole reaction volume was then transferred into an RNeasy Mini spin column, followed by RNA cleanup according to the manufacturer’s instructions. The obtained RNA was eluted into 40 μl of molecular biology-grade water and stored at −80°C. The quality and quantity of obtained RNA were assessed with an Agilent RNA 6000 Nano Kit (Agilent Technologies, DE), in combination with a P330 NanoPhotometer (Implen, DE). Only RNA samples with an RNA Integrity Number (RIN) >7 were used for further analysis. Residual DNA was digested with 2.4 U of TURBO DNase (Thermo Fisher Scientific, United States) in a 50 μl reaction containing a total of 2 μg of nucleic acid. The completeness of DNA digestion was verified by qPCR using a set of primers targeting the 16S rRNA gene, only samples with *c_p_* values corresponding to the non-template control were included in further analyses. Reverse transcription was performed with 200 U of M-MuLV Reverse Transcriptase (New England Biolabs, United States), in a 20 μl reaction containing 150 ng of total RNA and 8 U of Murine RNase inhibitor (New England Biolabs, United States), according to the manufacturer’s protocol.

Quantitative PCR was performed in a CFX Connect Real-Time PCR Detection System (Bio Rad, United States) employing a KAPA SYBR FAST qPCR Master Mix (2x) Kit (KAPA Biosystems, United States), in a total volume of 12 μl containing 0.25 μM primers targeting the gene *bphA* (forward primer 5'-GAGATCCAGAAGGGGCTAC, reverse primer 5'-GCGCATCCAGTGGTGATAC) or the reference 16S rRNA gene (forward primer 5'-GGATTAGATACCCTGGTA, reverse primer 5'-CCGTCAATTCATTTGAGTTT), with the addition of 0.5 μl of cDNA obtained in the previous step. The reaction conditions were as follows: (1) 95°C for 7 min; (2) 95°C for 20 s; (3) 60°C for 30 s; and (4) 72°C for 20 s, and 35 and 31 cycles were used for the *bphA* and 16S rRNA gene amplification, respectively. Each sample was run in triplicate. Non-template control was represented by the use of molecular biological water instead of the template. Specificity of amplification by all primer sets used in this study was determined by melting curve analysis of qPCR products and Sanger sequencing (data not shown).

The obtained *c_p_* values were processed by the Common Base Method according to Ganger et al. (2017), thus the level of *bphA* transcripts in a sample was normalized by the level of 16S rRNA copies. The induction of the *bphA* gene by the SPMs was expressed relative to the non-induced control, i.e., bacterial culture incubated without any amendment. Thus, the basal background transcription of the *bphA* gene corresponds to an induction rate of 1 in the presented data. The induction rates were tested with respect to the difference from the control (no inducer added) by multiple *t* tests with *p* values adjusted by the BH procedure to control the false discovery rate (Benjamini and Hochberg, 1995) at the level of 5%.

## Results

### Utilization of SPMs and Their Transformation by JAB1 and JAB1ΔbphA

**Figure 3:**
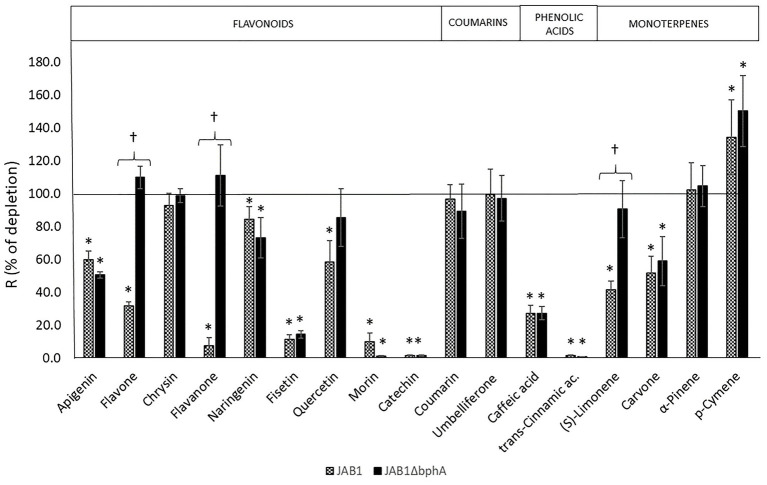
Degradation of secondary plant metabolites (SPMs) by strains JAB1 and JAB1ΔbphA. The ability of strains to degrade SPMs was tested by resting cell assay (RCA), depletion rates R in % of the corresponding abiotic controls are shown. Cells were incubated for 24 h in the presence of individual SPMs (0.25 mM). Error bars represent SD from three replicates. Asterisks (^∗^) show a depletion rate significantly different from the abiotic control, daggers (^†^) indicate a depletion by the wild-type JAB1 that is significantly different from the strain JAB1ΔbphA. One-way ANOVA (*p* < 0.05) was used to analyze the data.

In order to be able to distinguish the involvement of the JAB1-borne BPDO in the transformation of SPMs, the strain JAB1ΔbphA was employed alongside the wild-type strain. None of the SPMs tested (Figure 2) supported the growth of the JAB1 strain under the conditions applied (data not shown). Nevertheless, both JAB1 and JAB1ΔbphA were found to degrade apigenin (to 60 and 50% of the abiotic control, respectively), naringenin (84 and 73%), fisetin (11 and 14%), quercetin (58 and 85%), morin (10 and 2%), catechin (2% for both strains), caffeic acid (27% for both strains), *trans*-cinnamic acid (2 and 1%), and (*R*)-carvone (52 and 60%; Figure 3). In contrast, flavone, flavanone, and (*S*)-limonene were depleted solely by the strain JAB1 to 32, 7, and 42% of the control, respectively, and their levels were unaffected by the JAB1ΔbphA strain (Figure 3). This indicates that their transformation is mediated by the JAB1-borne BPDO. Chrysin, coumarin, umbelliferone, and α-pinene were neither degraded by JAB1 nor JAB1ΔbphA (Figure 3).

### Degradation Products of Flavone, Flavanone, and (*S*)-Limonene

**Figure 4:**
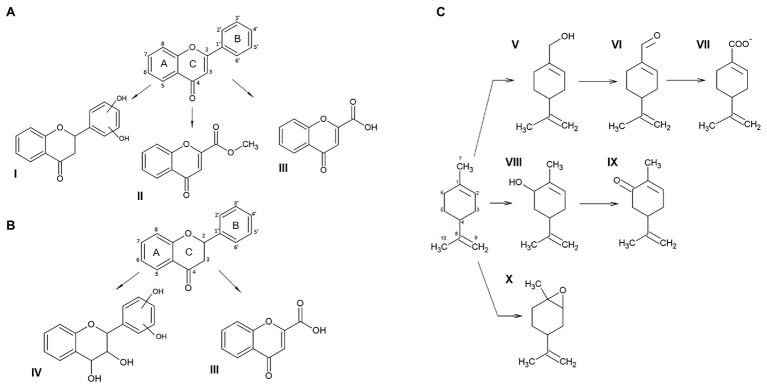
Products of flavone **(A)**, flavanone **(B)**, and (S)-limonene **(C)** degradation mediated by the JAB1-borne biphenyl 2,3-dioxygenase (BPDO). Compounds: I, 2-(m,n-dihydroxyphenyl)chromane-4-one; II, methyl 4-oxo-4H-chromene-2-carboxylate; III, 4-oxo-4H-chromene-2-carboxylic acid; IV, 2-(m,n-dihydroxyphenyl)chromane-3,4-diol; V, perillyl alcohol; VI, perillyl aldehyde; VII, perillic acid; VIII, carveol; IX, carvone; and X, limonene 1,2-epoxide.

To shed light on the mechanism of the degradation of flavone, flavanone, and (*S*)-limonene by JAB1, an analysis of their degradation products was performed. Three degradation products of flavone were found in the chromatogram of the extracts from the JAB1’s RCA reactions that were not present in the corresponding JAB1ΔbphA extracts (Supplementary Table S2; Supplementary Figure S2). The three compounds were identified as 4-oxo-4H-chromene-2-carboxylic acid (compound III in Figure 4A; Supplementary Table S2; Supplementary Figure S2), methyl 4-oxo-4H-chromene-2-carboxylate (compound II in Figure 4A; Supplementary Table S2), and 2-(m,n-dihydroxyphenyl)chromane-4-one (compound I in Figure 4A; Supplementary Table S2; Supplementary Figure S2). Analogously, two products of flavanone degradation by JAB1 were detected, which were identified as flavone 4-oxo-4H-chromene-2-carboxylic acid and 2-(m,n-dihydroxyphenyl)chromane-4-one (compounds III and IV, respectively, in Figure 4B; Supplementary Table S3; Supplementary Figure S2). Finally, six different products of JAB1-mediated (*S*)-limonene degradation were found and identified as perillyl alcohol (compound V in Figure 4C; Supplementary Table S4; Supplementary Figure S2), perillyl aldehyde (VI), perillic acid (VII), carveol (VIII), carvone (IX), and limonene 1,2-epoxide (X). Perillyl aldehyde and perillic acid were the major compounds in terms of quantity, whereas the levels of limonene-1,2-epoxide were the lowest, close to the limit of detection of the method (Supplementary Table S4).

### Induction of the *bphA* Gene in the JAB1 Strain by SPMs

**Figure 5:**
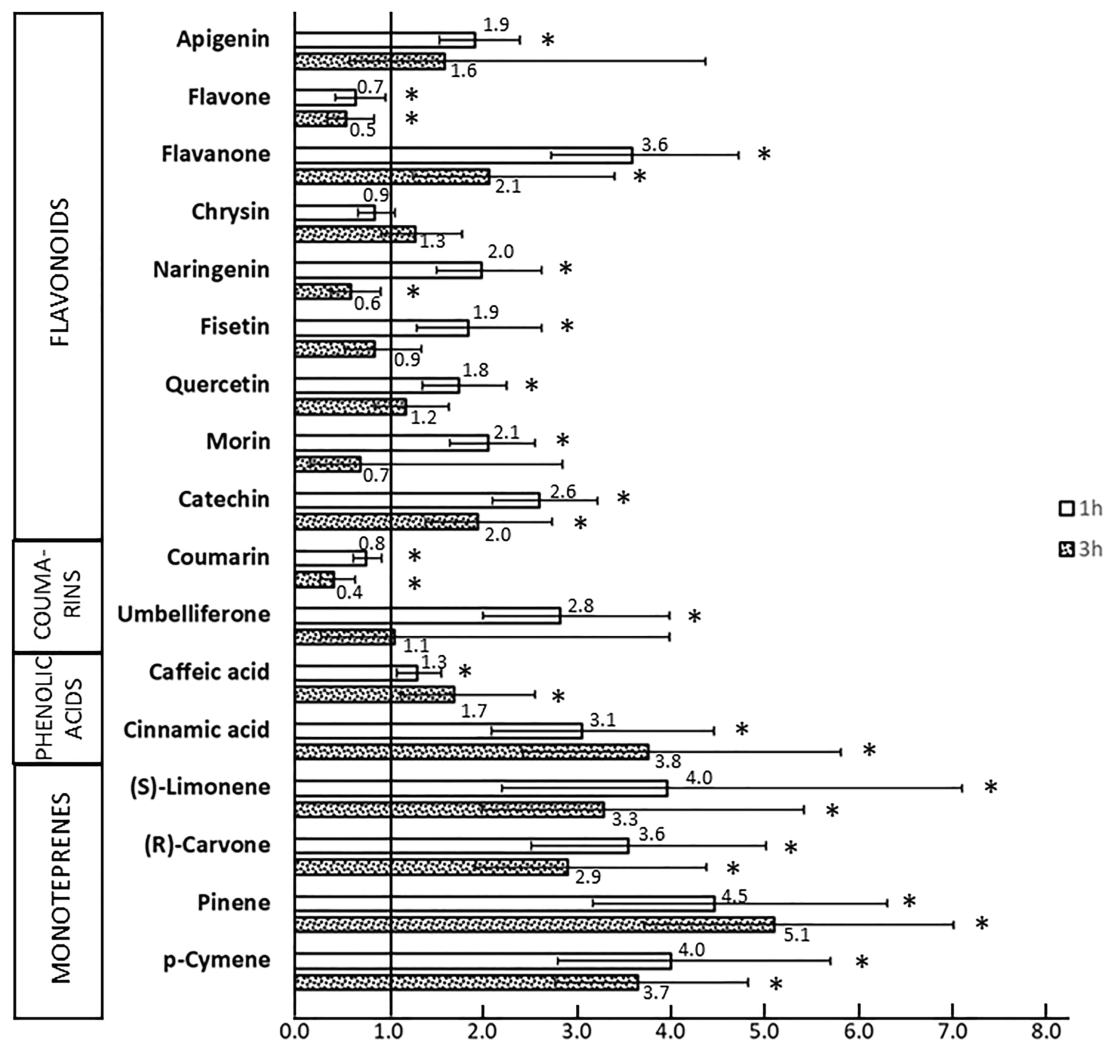
Relative levels of *bphA* transcripts in JAB1 strain exposed to SPMs. JAB1 cells were exposed to individual 0.25 mM SPMs for 1 and 3 h; the level of *bphA* transcripts was quantified by quantitative reverse transcription-PCR (RT-qPCR). The 16S rRNA transcript was used as a reference. The levels of *bphA* transcripts are shown as the average fold change in the non-induced control (cells without SPMs added, induction rate equals 1) with 95%-confidence intervals. The obtained *c*_p_ values were processed by the common base method according to Ganger et al. (2017). Asterisks (^∗^) denote a significant difference from the control as tested by *t* test (*α* = 0.05).

The ability of SPMs to induce the *bphA* gene in the JAB1 strain was tested by RT-qPCR; relative induction rates after 1 and 3 h of incubation with individual SPMs are shown in Figure 5. Seven out of the nine tested flavonoids, namely apigenin, catechin, fisetin, flavanone, morin, naringenin, and quercetin, increased the levels of *bphA*-transcripts after 1 h of co-incubation, ranging from a 1.8 (quercetin) to 3.6-fold (flavanone) induction of the non-induced control (no SPM added; Figure 5). Upon 3 h of incubation, these induction rates generally decreased (Figure 5). With flavone, a significant decrease in *bphA* transcripts compared to the control was observed at both time points, reaching up to 0.5-fold of the control after 3 h (Figure 5).

Upon the incubation of JAB1 cells with coumarin, *bphA*-transcript levels were significantly lowered compared to the non-induced control (by 0.4–0.8-fold of the control); in contrast, exposure to umbelliferone (7-hydroxycoumarin) resulted in *bphA* induction (2.8-fold, Figure 5). Two tested phenolics, *trans*-cinnamic acid and caffeic acid, increased the level of *bphA* transcripts after both 1 h (3.1 and 1.3-fold, respectively) and 3 h (3.8 and 1.7-fold respectively) of incubation (Figure 5).

Among the SPMs tested, monoterpenes were the strongest inducers of *bphA* in JAB1. Upon 1 h of exposure to all the monoterpenes tested, namely (*S*)-limonene, α-pinene, *p*-cymene, and (*R*)-carvone, the levels of *bphA* transcripts were approximately 4-fold of the non-induced control (Figure 5). After 3 h, the levels decreased to approximately 3-fold of the control for (*S*)-limonene, *α*-pinene, *p*-cymene, and (*R*)-carvone. The levels of *bphA* transcripts upon exposure to biphenyl, a common laboratory model inducer of the biphenyl pathway, culminated after 1 h incubation (9.6-fold of the control), and followed by a decrease to 2.1-fold at 3 h.

## Discussion

Pseudomonads have been reported to degrade a wide spectrum of aromatic pollutants, such as halogenated biphenyls, benzene, toluene, ethylbenzene, xylene, polyaromatics, dioxins, or DDT (Kimura et al., 1996; Baldwin et al., 2000; Ishiguro et al., 2000; Kamanavalli and Ninnekar, 2004; Uhlik et al., 2012; Wald et al., 2015). Generally, the aerobic degradation of such compounds is initiated by ARHDs. These enzymes incorporate two hydroxyl groups on the vicinal C-atoms of the aromatic ring, resulting in its activation for subsequent fission (Kweon et al., 2008). ARHDs have been reported to have broad substrate specificity, thus they are generally able to attack multiple structurally similar pollutants at the same time (Baldwin et al., 2000; Kweon et al., 2008). Apart from their involvement in the degradation of aromatic pollutants, ARHDs have been proposed to mediate the transformation of various compounds of natural origin, including SPMs (Focht, 1995; Pham et al., 2012). Indeed, in several studies, various bacterial ARHDs have been reported to catalyze the transformation of certain SPMs, including multiple flavonoids and mono- and diterpenes (Aoki et al., 1996; Eaton, 1996; Martin and Mohn, 1999; Kim et al., 2003; Seo et al., 2011a; Pham et al., 2012; Groeneveld et al., 2016; Agullo et al., 2017). Moreover, SPMs have been reported to induce ARHD activity (Park et al., 1999; Master and Mohn, 2001; Pham et al., 2015; Agullo et al., 2017). Based on all these studies, ARHDs have been hypothesized to also sustain environmental processes other than the degradation of aromatic pollutants (Pham et al., 2012; Pham and Sylvestre, 2013). In the soil environment, these processes could include the utilization of plant-derived compounds and the quenching of antimicrobial or signal compounds (Shaw et al., 2006; Cesco et al., 2010).

In our study, we aimed to provide a more complex insight into the involvement of the BPDO in the metabolism of plant-derived compounds in the soil bacterium *P. alcaliphila* JAB1. In its genome (Ridl et al., 2018), the strain JAB1 bears a single chromosome-encoded copy of the *bph* operon with architecture analogous to that of the model PCB-degrader *P. furukawaii* KF707 (Taira et al., 1992; Triscari-Barberi et al., 2012). Moreover, the predicted amino acid (aa) sequence of BphA from the strain JAB1 is identical to that from KF707, which was reported to mediate the transformation of flavone, flavanone, isoflavone, and isoflavanol (Harborne and Williams, 2000; Kim et al., 2003; Han et al., 2005; Seo et al., 2011b). The presence of a single copy of the *bphA* gene in the strain JAB1 enabled us to “switch off” the BPDO activity *via* a single site-specific recombination step (Figure 1). For that purpose, a lambda Red recombination system, originally designed for *P. aeruginosa*, was employed (Lesic and Rahme, 2008). The resulting strain JAB1ΔbphA and the wild-type strain were then used to assess the BPDO-mediated degradation of a range of SPMs including flavonoids, phenolic acids, coumarins, and monoterpenes (Figure 2). Although none of the SPMs supported the growth of JAB1, we demonstrated that the JAB1 strain is capable of degrading a wide range of aromatic SPMs, including flavonoids, monoterpenes, and phenolic acids (Figure 3). Importantly, the degradation of flavone, flavanone, and (*S*)-limonene by the JAB1 strain was found to be dependent on the presence of a functional BPDO (Figure 3). We therefore investigated the fate of these three compounds in JAB1 through the analysis of their degradation products.

Based on the analysis of flavone and flavanone degradation products mediated by the JAB1-borne BPDO (Figures 4A,B), we suggest that the enzyme attacked the B-ring of both flavone and flavanone, yielding 2-(m,n-dihydroxyphenyl)chromane-4-one. However, we were not able to determine the precise position of the hydroxylated carbons. The assumption that the dihydroxylation takes place on the B-ring is strongly supported by another structure identified among the degradation products of both flavone and flavanone, 4-oxo-4H-chromene-2-carboxylic acid, and, additionally, the by-product of flavone transformation, methyl-4-oxo-4H-chromene-2-carboxylate (Figures 4A,B). These structures support the scenario that the BPDO mediates initial dihydroxylation of the B-ring of flavone and flavanone, which is followed by subsequent reactions leading to its degradation. Attack on the unsubstituted B-ring of flavonoids (flavone, isoflavone, flavanone, and isoflavonol) was also reported for the BPDO of *P. furukawaii* KF707, producing 2',3'-dihydrodiols or 2',3'-epoxides (Kim et al., 2003; Han et al., 2005). In another study, the hybrid BPDO that had arisen from the DNA shuffling between *bphA* genes from *P. furukawaii* KF707 and *Paraburkholderia xenovorans* LB400 was shown to mediate the 2',3'-dihydroxylation and 3'-monohydroxylation of both flavone and flavanone, along with the 2'-monohydroxylation of flavanone (Chun et al., 2003). Further degradation of the hydroxylated flavonoid structures, resulting in the products identical or analogical to 4-oxo-4H-chromene-2-carboxylic acid found here, was proposed to be mediated by the rest of the biphenyl degradation pathway in *Par. xenovorans* LB400, *Pandoraea pnomenusa* B-356 (Pham et al., 2012), and *Rhodococcus erythropolis* U23A (Toussaint et al., 2012; Pham et al., 2015). Apart from flavone and flavanone, the transformation of which was shown to be BPDO-dependent, other flavonoids were found to be depleted by both the JAB1 wild-type and *bphA*-null strains, and therefore presumably transformed by enzymes other than BPDO (Figure 3).

The JAB1 strain was also found to be able to transform monoterpenes. Besides (*R*)-carvone that was depleted by both wild-type JAB1 and the *bphA*-null mutant, the degradation of (*S*)-limonene was demonstrated to depend on the presence of BPDO (Figure 3). The analysis of (*S*)-limonene degradation products in JAB1 suggests that the transformation is initiated by the monooxygenase activity of the BPDO targeting the C7 or C6 of the substrate, yielding perillyl alcohol and carveol, respectively. These products are further dehydrogenated into perillyl aldehyde (and eventually perillic acid) and carvone, respectively (Figure 4C). We therefore conclude that, apart from the dioxygenase activity, JAB1-borne BPDO is capable of mediating monooxygenation reactions, as was reported for, for instance, the naphthalene dioxygenase from *Pseudomonas* sp. strain NCIB 9816 (Resnick et al., 1996) or toluene dioxygenase from *P. putida* F1 (Robertson et al., 1992). Analogously, the transformation of monoterpenes, including (*S*)-limonene, by monooxygenation was also reported for the putative ARHD from *Pseudomonas* sp. PWD32 (Groeneveld et al., 2016). The hydroxylation of the C7 was also reported for the limonene dehydrogenase (CtmAB) from the betaproteobacterium *Castellaniella defragrans* 65Phen (Puentes-Cala et al., 2018). To rule out the possibility that a homologous enzyme would be responsible for the reaction in JAB1, the CmtA and CmtB protein sequences (Genbank accession no. CDM25290 and CDM25289, respectively) were blasted against the JAB1 complete genome but their orthologs were not identified. The hydroxylation of limonene at the C6 yielding carveol, which was further dehydrogenated into carvone, was also reported in *Rhodococcus opacus* PWD4 (Duetz et al., 2001). The degradation of (*S*)-limonene *via* perillyl alcohol and perillyl aldehyde, yielding perillic acid, was also demonstrated in the (*S*)-limonene-utilizing bacteria *Geobacillus stearothermophilus* BR388 (Cheong and Oriel, 1994), *P. gladioli* (Cadwallader et al., 1989), or in multiple fungi (Prema and Bhattacharyya, 1962; Noma et al., 1992; van Rensburg et al., 1997). Perillic acid as the product of (*R*)-limonene degradation was also found in *P. putida* GS1 (Speelmans et al., 1998). Another limonene-utilizing bacterium *Rhodococcus erythropolis* DCL14 was shown to employ the flavoprotein limonene 1,2-monooxygenase, which catalyzes the formation of 1,2-epoxy limonene (van der Werf et al., 1999). It is worth noting that a minute amount of 1,2-epoxy limonene was also detected in both the JAB1 wild-type and *bphA*-null strains (Supplementary Table S4), nevertheless, no homologue of the *R. erythropolis* DCL14 limonene 1,2-monooxygenase was found in the JAB1 genome (Ridl et al., 2018).

Additionally, we aimed to elucidate the role of SPMs in the regulation of the BPDO in the strain JAB1 as a representative of the KF707-type of the *bph* operon widely found in pseudomonads (Taira et al., 1992; Furukawa et al., 2004). In JAB1, a putative GntR-type transcription regulator encoded by the gene *gntR* found upstream of the *bphA* gene in JAB1 (Figure 1) shares 100% identity with the BphR1 from KF707 (formerly Orf0; Watanabe et al., 2000; Ridl et al., 2018). BphR1 was reported to act as the transcriptional activator involved in the upregulation of the *bph* operon promoted by 2-hydroxy-6-oxo-6-phenylhexa-2,4-dienoate (HOPDA), the product of BphC enzyme activity (Watanabe et al., 2000). Although the involvement of GntR-type regulators in the degradation of aromatic compounds is considered to be rather rare, members of this family were also found in several *bph* operons (Furukawa and Fujihara, 2008; Durante-Rodríguez et al., 2018); these include negative regulators BphS on the transposon Tn4371 from *Ralstonia eutropha* A5 (Mouz et al., 1999) and *Pseudomonas* sp. KKS102 (Ohtsubo et al., 2000). Moreover, the regulation of transcription of the *bph* operon in KF707 was found to be two-tiered. The transcription of both *bphR1* and structural *bph* genes in KF707 is promoted by another regulator LysR-type BphR2 encoded outside of the *bph* region that binds to the operators of *bphR1* and *bphAEFGBC* in response to the presence of biphenyl (Watanabe et al., 2003). When BphR2 homologue was searched for in the JAB1 genome, an ORF was found (positions 1,539,377–1,540,279 in the annotated genome sequence) outside of the *bph* cluster, the deduced protein sequence of which (300 aa, GenBank accession no. APU29489.1) shares 98.67% identity with BphR2. Therefore, based on the sequence and structural homology of their *bph* clusters and the presence of homologues of both regulator genes *bphR1* and *bphR2*, we assume that the transcription of the *bph* operon in JAB1 is analogous to that reported for KF707. Despite the *bphA* induction rates by biphenyl, a model laboratory substrate of BPDOs and inducer of *bph* operons, being the highest, we found that a wide range of SPM structures induced the *bphA* gene in JAB1 (Figure 5). Flavonoids that induce the *bphA* gene in JAB1 include the non-substituted flavone and flavanone, the flavone hydroxyderivative apigenin, flavonols fisetin, quercetin, morin, and the flavanol catechin (Figure 5). The decrease in *bphA*-transcript levels after 3 h of co-incubation observed for all the inducing flavonoids can be explained by their depletion through the activity of JAB1 cells. Indeed, all the flavonoids inducing the *bphA* gene in JAB1 were also degraded by this strain (Figures 3, 5). An analogous trend was also observed here for biphenyl (data not shown).

Since the chromone moiety was identified among flavone and flavanone degradation products, namely in the 4-oxo-4H-chromene-2-carboxylic acid (Figures 4A,B), we also tested the effect of coumarin and umbelliferone, i.e., structural analogues of chromone, on *bphA* transcription. Coumarin and umbelliferone differ only in the presence of the C7-hydroxyl group, and neither of them was degraded by the strain JAB1 (Figure 3). Despite this, umbelliferone acted as the inducer of *bphA* transcription, whereas exposure to coumarin even led to a decrease in *bphA*-transcript levels (Figure 5). Apparently, the presence of C7-hydroxyl in the umbelliferone structure seems to play a key role in the induction of *bphA* in JAB1.

The *bphA*-gene induction by the tested phenolic acids, specifically caffeic and cinnamic acid, was observed at both time points without a decreasing trend, even though they were both degraded by JAB1. Hence hypothetically, the induction effect of caffeic and cinnamic acid could be also attributed to the accumulated dead-end products of their degradation in JAB1. Nevertheless, the products of the JAB1-mediated degradation of caffeic and cinnamic acid were not analyzed within this study.

Furthermore, we demonstrated here that α-pinene, *p*-cymene, (*R*)-carvone, and (*S*)-limonene induced the *bphA* gene in the JAB1 strain. The levels of *bphA* transcripts in the presence of (*S*)-limonene, α-pinene, (*R*)-carvone, and *p*-cymene after 3 h of incubation were even higher than those of the model inducer biphenyl. Presumably, with (*S*)-limonene, such an observation could be attributed to the emergence and accumulation of carvone as the (*S*)-limonene degradation product (Figure 4C). Analogously, the upregulation of the *bph* degradation pathway is mediated by the biphenyl-degradation intermediate HOPDA (Ohtsubo et al., 2001; Watanabe et al., 2003). We therefore expect that in the JAB1 strain, the upregulation of the *bphA* gene observed for multiple flavonoids and terpenes is mediated either by the inducer *per se* or by intermediates of their degradation. Nevertheless, experiments that would provide evidence to support this hypothesis were beyond the scope of this study.

SPMs, either in the form of root exudates or artificial mixtures, were already reported to promote the degradation of PCBs (Donnelly et al., 1994; Focht, 1995; Gilbert and Crowley, 1997, 1998; Hernandez et al., 1997; Singer et al., 2000; Tandlich et al., 2001; Toussaint et al., 2012). Nevertheless, studies that would elucidate a molecular basis of this phenomenon have been relatively scarce. A few pieces of evidence showed, for instance, that the monoterpene carvon acts as the inducer of the *bphC* gene in *Cupriavidus necator* H850 (Park et al., 1999). On the other hand, the study of Master and Mohn (2001) showed a negative effect of SPMs on the *bphA* expression in *Pseudomonas* sp. Cam-10, including monoterpenes (carvone, cumene, limonene, and cymene) and the flavonoid myricetin. More recently, Pham et al. (2015) reported multiple flavonoids to induce the activity of the biphenyl pathway in *R. erythropolis* U23A. The induction of *bphA* genes by various SPMs observed in that study as well as in this report suggests that such an upregulation is an adaptive trait, and not an experimental artifact or only a consequence of a leakiness of regulatory mechanisms caused by their low signal specificity. Our research, which is the first more complex report which takes into account multiple classes of SPMs and investigates their ability to induce and act as substrates of BPDO, supports the hypothesis that the ability of BPDO to degrade aromatic pollutants might have evolved from its ability to degrade SPMs. While this ability may have been weakened in bacterial isolates evolving in a contaminated environment, the inductive effects of SPMs have remained. This prediction, however, requires further investigations.

In summary, our results are in line with the previous findings of Pham et al. (2012), who suggested that bacterial BPDOs serve ecological roles other that the degradation of anthropogenic aromatic pollutants. These roles should specifically include the transformation of SPMs, a process with multiple implications for the fate of soil plant-derived carbon fluxes and signaling. Our study demonstrates the capacity of the JAB1 strain to transform a wide range of SPMs and indicates the possible involvement of JAB1’s BPDO in soil processes mediated by SPMs, namely flavonoids and terpenoids. Presumably, besides its potential role in the attenuation of PCB contamination in the soil of origin, the BPDO in JAB1 could participate in the protection of the bacterium against the selective pressure caused by these specific SPMs through their degradation (Falcone Ferreyra et al., 2012; Górniak et al., 2019; Huang and Osbourn, 2019). With this in mind, more research needs to be conducted to disclose the role of the *bph* pathway in the response to plant-derived compounds in the background of the real soil environment.

## Data Availability Statement

The raw data supporting the conclusions of this article will be made available by the authors, without undue reservation.

## Author Contributions

AZ, JSum, and OU planned and designed the research and wrote the manuscript. AZ, KM, JSem, MS, TC, and JSum performed experiments and/or analyzed data. All authors contributed to the article and approved the submitted version.

### Conflict of Interest

The authors declare that the research was conducted in the absence of any commercial or financial relationships that could be construed as a potential conflict of interest.

## Abbreviations

aa: Amino acid
ARHD: Aromatic ring-hydroxylating dioxygenases.
BPDO: Biphenyl 2,3-dioxygenase
HOPDA: 2-Hydroxy-6-oxo-6-phenyl-2,4-hexadienoic acid
RCA: Resting cell assay
PCBs: Polychlorinated biphenyls
SPMs: Secondary plant metabolites.
