# Supplementary material for: Biphenyl 2,3-Dioxygenase in Pseudomonas alcaliphila JAB1 Is Both Induced by Phenolics and Monoterpenes and Involved in Their Transformation
Source: Front Microbiol. 2021 Apr 30;12:657311. doi: 10.3389/fmicb.2021.657311 (PMC8119895; doi:10.3389/fmicb.2021.657311)
Supplement: Supplementary file 1 [file Data_Shee_1.pdf]

SUPPLEMENTARY INFORMATION

Biphenyl 2,3-dioxygenase in *Pseudomonas alcaliphila* JAB1 is both induced by phenolics and monoterpenes and involved in their transformation

Andrea Zubrova[1], Klara Michalikova[2], Jaroslav Semerad[2], Michal Strejcek[1], Tomas Cajthaml[2,3], Jachym Suman[1✉], Ondrej Uhlik[1✉]

[1]*University of Chemistry and Technology, Prague, Faculty of Food and Biochemical Technology, Department of Biochemistry and Microbiology, Prague, Czech Republic*

[2]*Institute of Microbiology, Academy of Sciences of the Czech Republic, v.v.i., Prague, Czech Republic*

[3]*Charles University, Faculty of Science, Institute for Environmental Studies, Prague, Czech Republic*

✉**Corresponding authors:** University of Chemistry and Technology, Prague, Technicka 3, 166 28 Prague 6, Czech Republic. Phone: +420 220 44 5136; email: ondrej.uhlik@vscht.cz (OU), jachym.suman@vscht.cz (JS)

Utilization of SPMs by the strain JAB1

The ability of the strain JAB1 to utilize the SPMs listed in Figure 2 as a sole carbon source was tested in liquid MSS. JAB1 culture was cultivated overnight (O/N) in LB, cells were harvested by centrifugation, washed twice in 0.85% NaCl and resuspended in MSS. The resulting cell suspension was used to inoculate MSS with SPM in Erlenmeyer flasks to reach a final optical density at 600 nm (OD_{600}) of 0.025. The stock solutions of the tested substrates were previously pipetted onto the bottom of Erlenmeyer flasks and ethanol was evaporated. The SPM concentration levels tested were 0.5, 1, and 3 $mmol.l^{-1}$. Upon inoculation, bacterial cultures were cultivated for 6 days at 28 °C/130 rpm. The cell growth was monitored by measuring OD_{600} (MSS-soluble substrates: flavanone, flavone, terpenoids, phenolic acids, coumarins), or by colony counting upon plating serial dilutions of 100 µl aliquots of the culture on LB agar plates (low-soluble substrates: apigenin, fisetin, chrysin, morin, naringenin, quercetin). The flasks bearing MSS with added substrates but without inoculation were used as abiotic references.

Construction of the JAB1 *bphA*-null mutant

For the purposes of plasmid cloning, an In-Fusion® HD Cloning Kit (ClonTech, USA) was employed. First, the pBluescript II KS(+)-based plasmid named herein pBS-neoR-F12 (*ampR* selection) was constructed, bearing a gene for neomycin phosphotransferase (*neoR*, amplified from the plasmid pDrive, Qiagen) flanked by ca 500 bp-long fragments of JAB1 chromosome located upstream and downstream of the *bphA* gene (Figure S1). The plasmid pBS-neoR-F12, bearing the recombination cassette for the replacement of the *bphA* gene in the JAB1 genome, was constructed in three consecutive steps using an In-Fusion® HD Cloning Kit (ClonTech, USA), as shown in Figure S1. The In-Fusion steps were performed according to the manual and used for the transformation of *E. coli* DH11S cells; transformants were selected on LB plates containing ampicillin 150 mg/l. All the PCR amplifications were performed using KAPA HiFi HotStart ReadyMix (Roche) using the primer combinations presented in Table S1. In the first step, the P_{lac}-*neoR* cassette amplified from pDrive (Qiagen) (primers neoRF and neoRR, Table S1) was inserted into the pBluescript II KS(+) plasmid backbone (amplified using primers pBSF and pBSR, Table S1), yielding the plasmid pBS-neoR. In the second step, a 500 bp-long DNA span here designated as Flank1, located upstream of the *bphA* gene in the JAB1 genome, was amplified from the JAB1 genomic DNA (primers flank1F and flank1R, Table S1) and inserted into the pBS-neoR backbone (amplified using pBSF and pBS-neoRR, Table S1), yielding pBS-neoR-F1. Finally, a 500bp-DNA span Flank2 located downstream of the *bphA* gene in the JAB1 genome was amplified using the primers flank2F and flank2R and inserted into the pBS-neoR-F1 (amplified using pBS-neoR-F1F and pBSR, Table S1), yielding pBS-neoR-F12 (Figure S1).

The plasmid pBS-neoR-F12 was then used as a template for the PCR amplification of the *neoR*-bearing recombination cassette F1-*neoR*-F2 intended for site-specific recombination in JAB1 (primers flank1F and flank2R, Table S1).

Further, to ensure efficient chloramphenicol-based selection of the respective JAB1 transformants, the *ampR* coding sequence in the original pUCP18-RedS was replaced with chloramphenicol acetyltransferase gene *catR* (amplified from the plasmid pQE-31, Qiagen), yielding pUCP18-RedS-catR (Figure S1). The plasmid pUCP18-RedS-catR was constructed employing an In-Fusion® HD Cloning Kit (ClonTech, USA). The *catR* (chloramphenicol acetyltransferase) coding sequence was amplified from the plasmid pQE-31 (Qiagen) using the primers catRF and catRR (Table S1) and fused with the pUCP18-RedS core, amplified using the primers pUCPF and pUCPR from puCP18-RedS (Lesic & Rahme, 2008) and lacking the original ampicillin resistance gene *ampR* (Figure S1). The *E. coli* DH11S transformants were selected on LB plates containing 50 mg/l chloramphenicol.

The resultant plasmid pUCP18-RedS-catR was then introduced into JAB1 cells via electroporation (Choi *et al.*, 2006), the transformants were selected on LB agar plates containing chloramphenicol (300 $mg.l^{-1}$). The JAB1 cells bearing pUCP18-RedS-catR were induced by 0.5% (w/v) l-arabinose for 1h and the purified (DNA Clean & Concentrator, Zymo Research) recombination cassette F1-*neoR*-F2 prepared as described above was introduced into the cells by electroporation (for details see Lesic and Rahme 2008). The recombinants were then selected on LB plates supplemented with kanamycin (10 $mg.l^{-1}$). The replacement of the *bphA* gene by *neoR* in individual kanamycin-resistant colonies was then verified by PCR amplification and subsequent Sanger sequencing of the portion of the JAB1 chromosome corresponding to the recombination cassette (Figure S1). Due to the replacement of the *bphA* coding sequence with the neomycin phosphotranferase gene (*neoR*), the strain JAB1ΔbphA had non-functional BPDO, hindering the utilization of biphenyl as a sole carbon source, as tested on solid MSS-based medium (data not shown). Simultaneously, the lack of BPDO activity in the recombinants was verified by their inability to utilize biphenyl as a sole carbon source. The retained 2,3-dihydroxybiphenyl 1,2-dioxygenase (BphC) activity in JAB1ΔbphA cells was assessed by the addition of 2,3-dihydroxybiphenyl (0.5mM) to the washed O/N culture in LB and observation of the evolution of the yellow meta-cleavage product 2-hydroxy-6-oxo-6-phenyl-2,4-hexadienoic acid (HOPDA) (data not shown) (Furukawa & Miyazaki, 1986; Hayase *et al.*, 1990).

Table S1: Primers used for the construction of the plasmid pBS-neoR-F12 and pUCP18-RedS-catR. Details on the construction are given in the Supplementary text.

**Primer designation**	**Sequence (5' → 3')**	**Amplified DNA fragment**	**Template DNA used**	**Annealing temperature [° C]**
**Construction of plasmid pBS-neoR**				
pBSF	AGCTGTTTCCTGTGTGAAATTG	Vector backbone	pBluescript II KS(+)	55
pBSR	GGTGATGGTTCACGTAGTGG			
neoRF	CCAATACGCAAACCGACCTCTCCCCGCGCGTTGGC	Insert (P_{lac}-*neoR* cassette)	pDrive (Qiagen)	50
neoRR	ACGTGAACCATCACCTTAGAAAAACTCATCGAGCATC			
**Construction of plasmid pBS-neoR-F1**				
pBSF	CGGTTTGCGTATTGGGCGCTC	Vector backbone	pBS-neoR	60
pBS-neoRR	ACCTCTCCCCGCGCGTTGGC			
flank1F	CCAATACGCAAACCGCCGGCGGTTCCTGTCCATCCTG	Insert (Flank1, 500 bp)	JAB1 genomic DNA	55
flank1R	CGCGCGGGGAGAGGTATGACTCTCGTGCCCAAGTAAC			
**Construction of plasmid pBS-neoR-F12**				
pBS-neoR-F1F	TTAGAAAAACTCATCGAGCATC	Vector backbone	pBS-neoR-F1	50
pBSR	GGTGATGGTTCACGTAGTGG			
flank2F	GATGAGTTTTTCTAATCAAGACGCAATCGTTAGATC	Insert (Flank2, 500 bp)	JAB1 genomic DNA	50
flank2R	ACGTGAACCATCACCAAGGTATCCGGCGTGGCCGTC			
**Construction of plasmid pUCP18-RedS-catR**				
catRF	TGAAAAAGGAAGAGTATGGAGAAAAAAATCACTG	Insert (*catR* coding sequence)	pQE-31 (Qiagen)	
catRR	TTTGATCTTTTCTACTTACGCCCCGCCCTGCCACTC			
pUCPF	GTAGAAAAGATCAAAGGATC	Vector core	pUCP18-RedS	
pUCPR	ACTCTTCCTTTTTCAATATTATTG			

A **B**

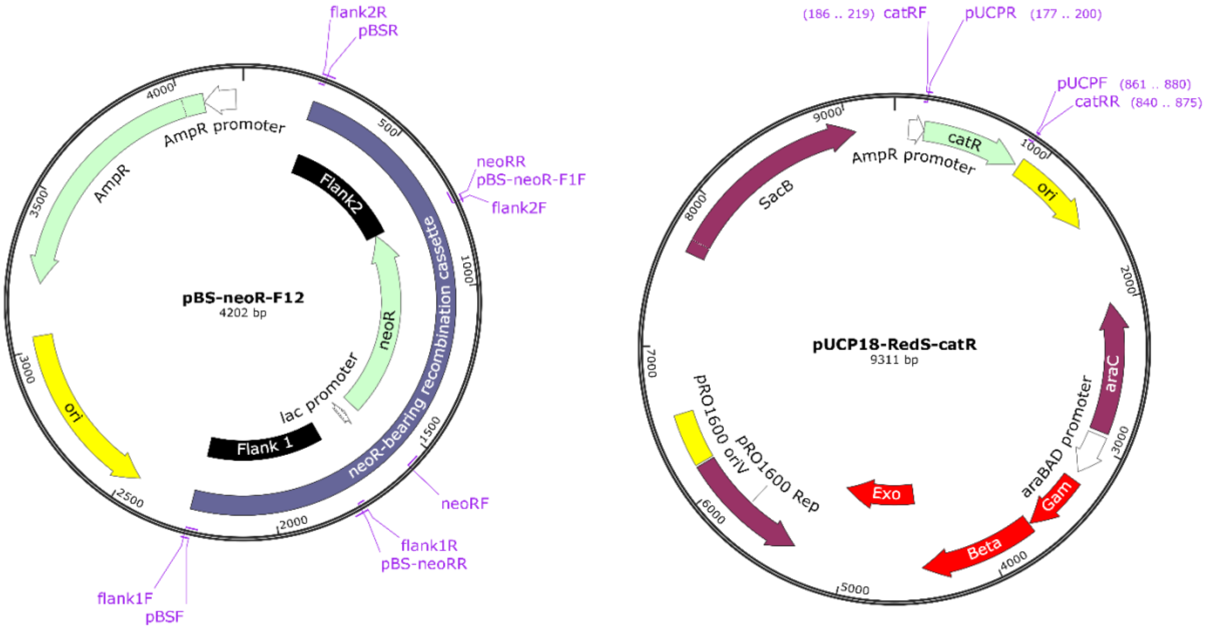

Figure S1: Maps of plasmids pBS-neoRF12 (A) and pUCP18-RedS-catR (B). The position of primers used for the construction (Table S1) is indicated. In (A), the DNA span bearing the recombination cassette used for *bphA* deletion in JAB1 is depicted.

Analysis of SPM depletion and degradation products

The samples listed in Figure 2, except those with flavone, flavanone and *p*-cymene, were extracted after RCA with an aliquot of ethyl acetate (5 ml; 3 times) using a reciprocal shaker. For the purposes of gas chromatography analyses, combined extracts of individual samples were dried with sodium sulfate, concentrated to approximately 1 ml using a nitrogen stream, and analyzed by GC-MS. Samples for LC-MS (QTRAP) and LC-UV analysis were diluted appropriately with methanol.

The analysis of the depletion of chrysin, apigenin, naringenin, fisetin, catechin, quercetin, morin, caffeic acid, umbelliferon and coumarin (Figure 3) was performed using a NexeraXR ultra-high performance liquid chromatograph (Shimadzu, Japan) coupled with a Qtrap 4500 mass spectrometer (Sciex, USA) with electrospray ionization (ESI) operating either in positive or negative mode. 5 µl of the sample was injected into a CortecsT3 2.7 µm, 3 mm x 150 mm chromatographic column (Waters, USA) with the corresponding guard column. The mobile phase was composed of water containing 0.1% formic acid and 10% acetonitrile (v/v) (A) and acetonitrile (B); the flow rate was 0.4 ml/min. The column temperature was maintained at 40 °C. Gradient elution was as follows (time [min]/% B): 0/20; 5/60; 7/60; 9/100; 11/100; 11.5/20; 15/20. Two specific ion transitions were monitored for each analyte in multiple-reaction monitoring mode (MRM): negative (Q1/Q3): chrysin 253/107;135, apigenin 269/117;135, naringenin 271/151/177, fisetin 285/135;163, catechin 289/245;109, quercetin 301/151;179, morin 301/151;125, caffeic acid 179/134;107 and umbelliferone 161/133;93); positive: coumarin 147/103;65. The conditions for the Turbo V^{TM} ion source were as follows: curtain gas: 30 psi, ion spray voltage: 5.5 kV positive mode/–4.5 kV negative mode, vaporizer temperature: 450 °C, ion source gas 1: 40 psi, ion source gas 2: 50 psi.

The analysis of the depletion of cinnamic acid (Figure 3) was performed with an Alliance 2695 liquid chromatograph (Waters, Milford, MA) equipped with a diode-array detector (Waters 2996). The analyte was separated in an XBridge C18 column (150 mm × 3.6 mm, particle diameter 3.5 µm; Waters) with a flow rate of 0.8 ml/min at 35 °C. A gradient protocol with a mobile phase containing 0.1% formic acid and 10% acetonitrile (v/v) (A) and acetonitrile (B) was performed (time/% B: 0/0; 2/0; 10/70; 11/0; 15/0). The detection wavelength for cinnamic acid was 275 nm.

Targeted and untargeted analysis of flavone (Table S2, Figure S2), flavanone (Table S3, Figure S2,) and *p*-cymene in the cell suspension was performed by using an Agilent 1260 Infinity II liquid chromatograph coupled to a high-resolution Agilent QTOF 6546 mass spectrometer – QTOF MS equipped with an Agilent Jet Stream electrospray ion source (Agilent Technologies, USA). The separation of analytes was performed in a Kromasil 2.5 µm, 2.1 mm x 10 mm chromatographic column (Azko Nobel, The Netherlands). The mobile phase was composed of 0.5 mM ammonium fluoride in water and methanol. The gradient elution started with 10% methanol for 0.5 min and increased to 100 % in 10 min, the column was equilibrated for 5 min

to the starting conditions. The flow rate was 0.3 $ml.min^{-1}$ and the column temperature was maintained at 40 °C. The volume of 1 µl of the sample was injected. The QTOF MS, operating in positive mode, was tuned using Swarm Autotune for the mass range, m/z 50–750. Purine (m/z 121.050873) was used as the reference ion during the analysis to achieve the best mass accuracy. A single MS mode (ESI+, 5 spectra/s) over the range 50 – 600 m/z was chosen to perform metabolite profiling on the cell extracts. Agilent MassHunter Profinder version B.08.00 was used for recursive molecular feature extraction. The resulting features were evaluated with Agilent MassHunter Profiler Professional 15.1 (MPP) using the filter on the volcano plot algorithm with a cutoff of $p < 0.05$ and fold change > 1.5. Differential features were annotated using the ID Browser tool in MPP. Unique features revealed in bacterial strain JAB1 samples were exported to the inclusion list of an autoMS/MS method and acquired using three different collision energies (CE = 10, 20, and 40 eV) and a fragmentor voltage of 140 V. The mass range was 50-600 m/z and the MS/MS acquisition rate was 5 spectra/s. Compound identification and structure clarification were accomplished using MS/MS spectra matching (Agilent Metlin MS/MS library) and for missing spectra matches, Agilent MassHunter Molecular Structure Correlator B.08.00 software was employed to assist the structure elucidation.

The depletion of monoterpenes ((*S*)-limonene, carvone, α-pinene) (Figure 3) and nontargeted analysis of (*S*)-limonene degradation products (Table S4, Figure S2) were analyzed with a Varian 450-GC instrument (USA) equipped with a Combi-Pal injector (CTC Analytics, Sweden) and a Varian 240 MS ion trap mass spectrometer employing electron ionization. The injection volume of the sample was 1 µl. Separation proceeded in a DB-5MS column (Agilent, Germany), 30 mm long x 0.25 mm I.D., 0.25 µm film thickness. The GC oven temperature program started from 45 °C (hold 6 min), then gradually increased to 80 °C (2 °C/min, hold 5 min), and finally heated up to 200 °C (10 °C/min), where it was held isothermally for 2 min. The GC oven temperature program for the analysis started from 60 °C (hold 1 min), then gradually increased to 280 °C (15° C/min, hold 10 min). Helium (99.999%) at a flow rate of 1 ml/min was used as the carrier gas. The injector temperature was 240 °C. The source, ion trap, and transfer line temperatures were 250, 220, and 280 °C, respectively. The mass spectra were recorded at 3 scan/s at 70 eV over the mass range 50 – 300 amu (50 – 500 amu in the case of nontargeted analysis). Possible hydroxylated metabolites were assessed after the derivatization of the sample. Ethyl acetate extract (1ml) was mixed with 200 µl of N,O-bis(trimethylsilyl)trifluoracetamide (BSTFA): trimethylsilane TMS (99:1, v/v) and incubated at 70 °C for 15 min. The GC oven temperature for the derivatized samples started from 60 °C (hold 1 min), then gradually increased to 120 °C (25 °C/min), and finally heated up to 240 °C (2.5 °C/min), which was held isothermally for 9 min.

Table S2. Products of flavone degradation in JAB1 strain. Proposed structures correspond with Figure 4A.

Proposed structure (compound no.)	Suggested formula	t_R (min)	m/z measured	m/z theoretical	m/z of fragment ions (relative intensity)
2-(m,n-dihydroxyphenyl)-chroman-4-one **(I)**	$C_{15}H_{12}O_4$	8.30	257.0807	257.0808	239.0720 (87), 229.0953 (22), 211.0752 (100), 131.0502 (17), 121.0288 (25)
methyl 4-oxo-4H-chromene-2-carboxylate **(II)**	$C_{11}H_8O_4$	8.50	205.0495	205.0495	205.0492 (100), 177.0545 (42), 135.0439 (32), 77.0386 (44)
4-oxo-4H-chromene-2-carboxylic acid **(III)**	$C_{10}H_6O_4$	4.34	191.0340	191.0339	191.0340 (100), 147.0440 (26), 121.0286 (6), 95.0491 (34), 77.0385 (43)

Table S3. Products of flavanone degradation in JAB1 strain. Proposed structures correspond with Figure 4B.

Proposed structure (compound no.)	Suggested formula	t_R (min)	m/z measured	m/z theoretical	m/z of fragment ions (relative intensity)
4-oxo-4H-chromene-2-carboxylic acid **(III)**	$C_{10}H_6O_4$	4.34	191.0340	191.0339	191.0340 (100), 147.0440 (26), 121.0286 (6), 95.0491 (34), 77.0385 (43)
2-(m,n-dihydroxyphenyl)-chroman-3,4-diol **(IV)**	$C_{15}H_{14}O_5$	6.28	275.0916	275.0914	275.0926 (100), 247.0958 (61), 229.0859 (43), 211.0771 (17), 183,0807 (17), 121.0283(68), 115.0403 (48), 87.0434 (56)

Table S4. Products of *(S)*-limonene degradation in JAB1 strain. Proposed structures correspond with Figure 4C.

Proposed structure (compound no.)	Trivial name	MW suggestion	Peak height	t_R (min)	m/z of fragment ions (relative intensity)
(4-(prop-1-en-2-yl)cyclohex-1-en-1-yl)methanol **(V)**	perillyl alcohol	152	5217	31.0	152 (1), 137 (6), 121 (60), 105 (48), 91 (68), 79 (100), 67 (48), 55 (27)
4-(prop-1-en-2-yl)cyclohex-1-ene-1-carbaldehyde **(VI)**	perillyl aldehyde	150	88102	35.8	95 (39), 94 (100), 79 (88), 67 (33)

4-(prop-1-en-2-yl)cyclohex-1-ene-1-carboxylic acid **(VII)**	perillic acid	166	19094	36.1	166 (24), 121 (35), 91 (42), 79 (78), 67 (100)
2-methyl-5-(prop-1-en-2-yl)cyclohex-2-en-1-ol **(VIII)**	carveol	152	3968	30.8	152 (1), 137 (10), 121 (41), 105 (40), 91 (82), 79 (100), 67 (33)
2-methyl-5-(prop-1-en-2-yl)cyclohex-2-en-1-one **(IX)**	carvone	150	16611	32.6	108 (51), 93 (87), 82 (100), 79 (47), 54 (46)
1-methyl-4-(prop-1-en-2-yl)-7-oxabicyclo[4.1.0]heptane **(X)**	limonene 1,2-epoxide	152	4039	31.7<br>31.8	135 (28), 94 (92), 79 (100), 67 (28)

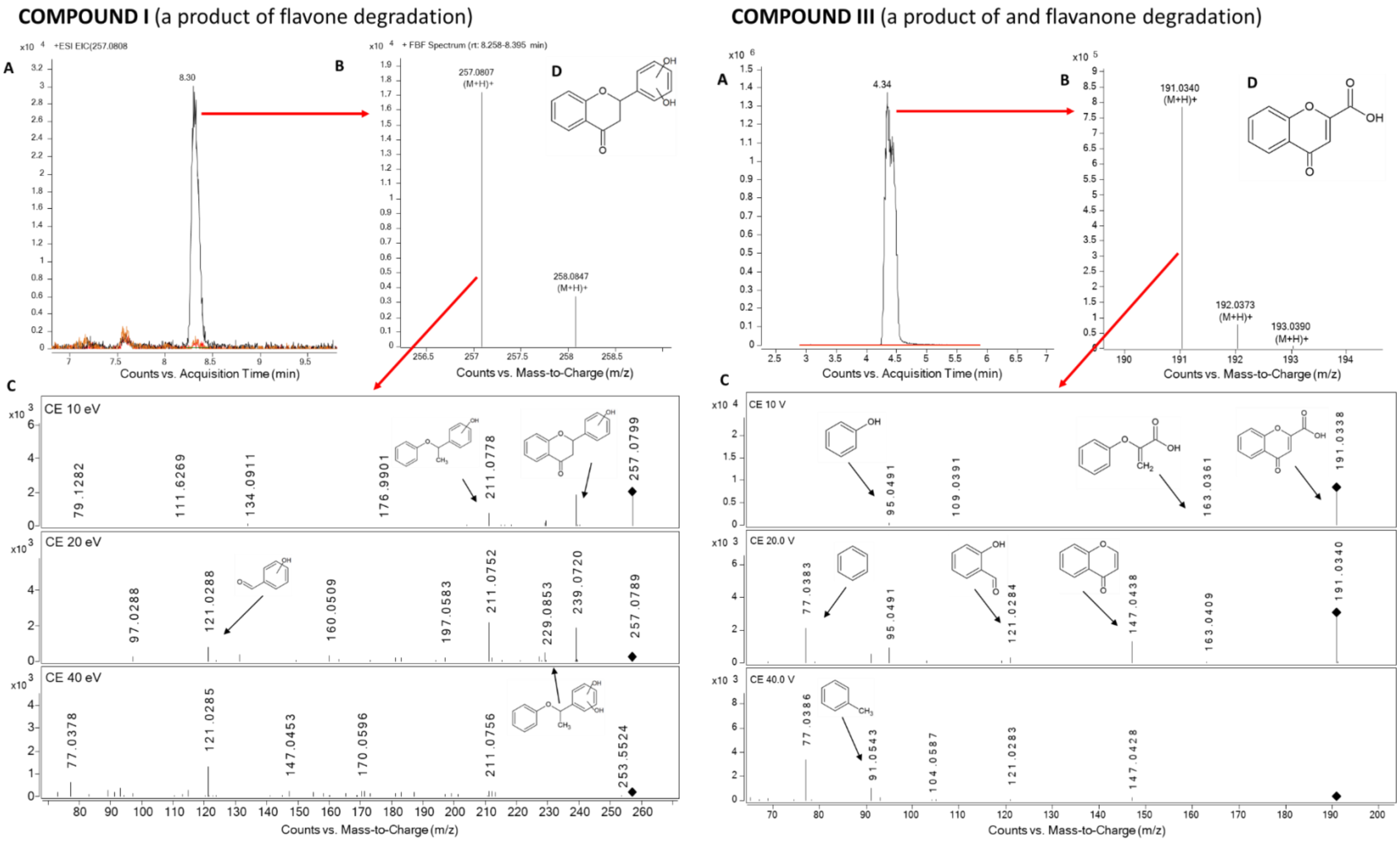

Figure S2: Analysis of degradation products of flavone, flavanone degradation (compounds I, III, and IV in Table S2 and S3), and (*S*)-limonene (compounds V- X in Table S4) in JAB1 strain with the contribution of BPDO. A, section of LC chromatogram (ESI+ mode); B, MS spectrum corresponding to peak in A; C, fragmentation MS spectra using different collision energies (CE); D, proposed structure.

COMPOUND IV (flavanone degradation)

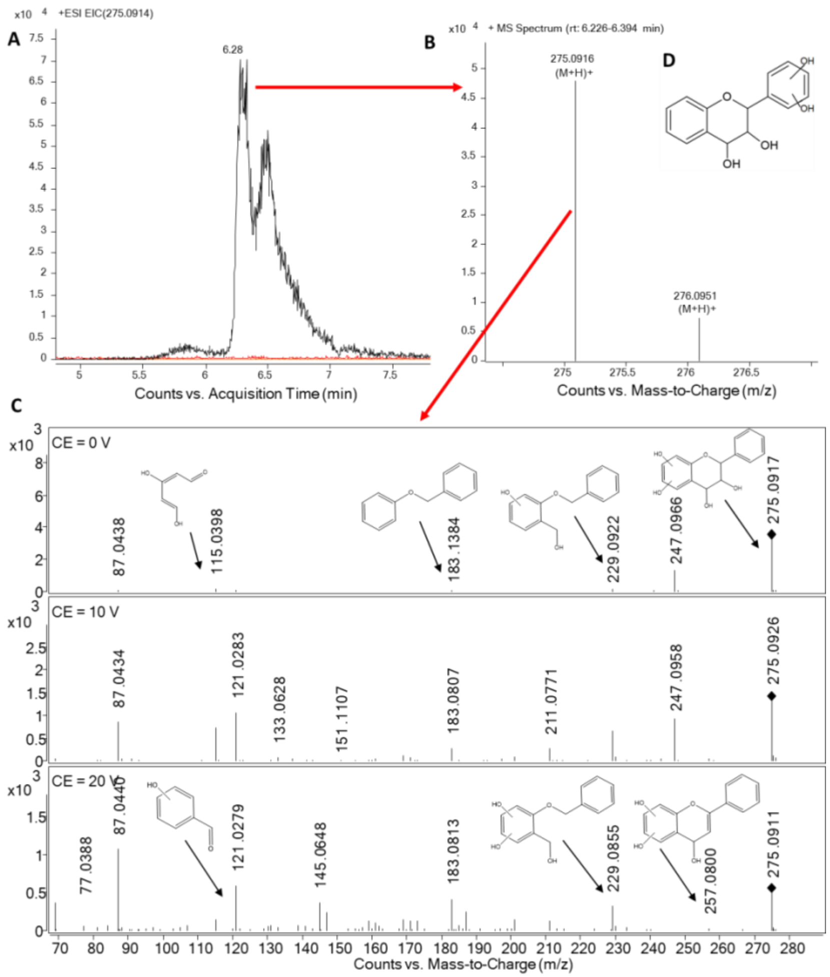

COMPOUNDS V- X [(*S*)-limonene degradation]

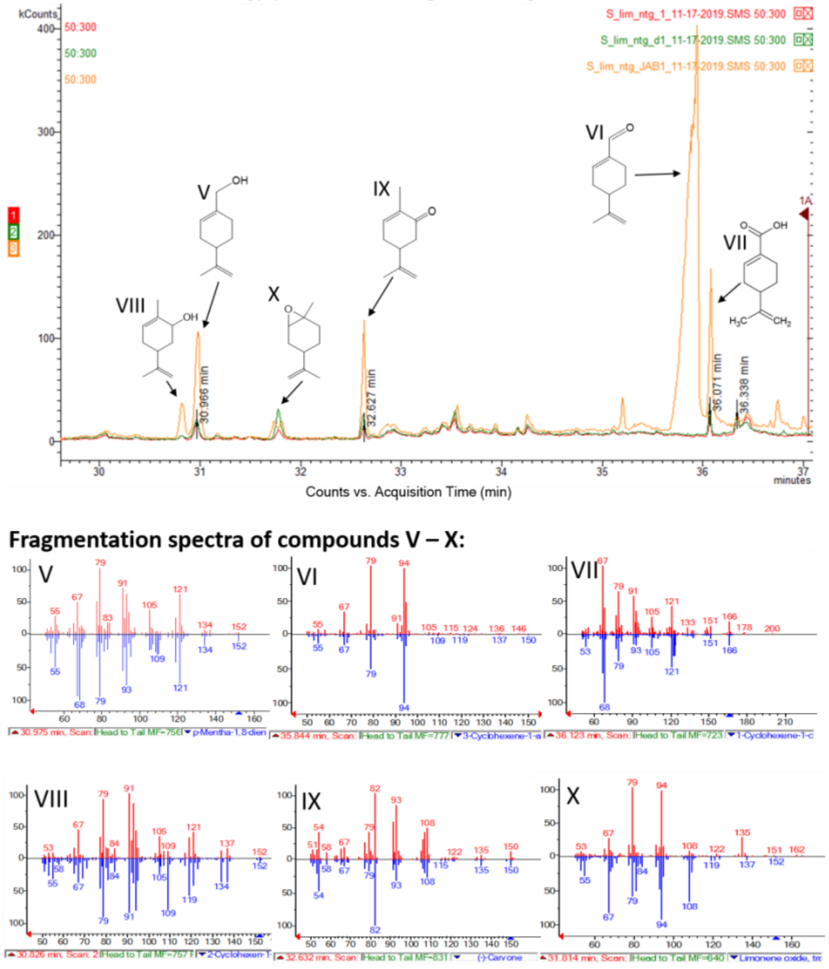

Figure S2 continued: Analysis of products of flavone, flavanone (compounds I, III, and IV in Table S2 and S3), and (*S*)-limonene (compounds V - X Table S4) degradation by the strain JAB1. A, section of LC chromatogram (ESI+ mode); B, MS spectrum corresponding to peak in A; C, fragmentation MS spectra using different collision energies (CE); D, proposed structure.

References

Choi KH, Kumar A, Schweizer HP. 2006. A 10-min method for preparation of highly electrocompetent Pseudomonas aeruginosa cells: application for DNA fragment transfer between chromosomes and plasmid transformation. *Journal of Microbiological Methods* **64**(3): 391-397.

Furukawa K, Miyazaki T. 1986. Cloning of a gene cluster encoding biphenyl and chlorobiphenyl degradation in Pseudomonas pseudoalcaligenes. *Journal of bacteriology* **166**(2): 392-398.

Hayase N, Taira K, Furukawa K. 1990. Pseudomonas putida KF715 bphABCD operon encoding biphenyl and polychlorinated biphenyl degradation: cloning, analysis, and expression in soil bacteria. **172**(2): 1160-1164.

Lesic B, Rahme LG. 2008. Use of the lambda Red recombinase system to rapidly generate mutants in *Pseudomonas aeruginosa*. *BMC Molecular Biology* **9**: 20-20.
